# Deposition chamber technology as building blocks for a standardized brain-on-chip framework

**DOI:** 10.1038/s41378-022-00406-x

**Published:** 2022-08-01

**Authors:** B. G. C. Maisonneuve, L. Libralesso, L. Miny, A. Batut, J. Rontard, M. Gleyzes, B. Boudra, J. Viera, D. Debis, F. Larramendy, V. Jost, T. Honegger

**Affiliations:** 1 University Grenoble Alpes, CNRS, LTM, 38000 Grenoble, France; 2 University Grenoble Alpes, CNRS, GSCOP, 38000 Grenoble, France; 3 NETRI, 69007 Lyon, France

**Keywords:** Microfluidics, Electrical and electronic engineering

## Abstract

The in vitro modeling of human brain connectomes is key to exploring the structure-function relationship of the central nervous system. Elucidating this intricate relationship will allow better studying of the pathological mechanisms of neurodegeneration and hence result in improved drug screenings for complex neurological disorders, such as Alzheimer’s and Parkinson diseases. However, currently used in vitro modeling technologies lack the potential to mimic physiologically relevant neural structures. Herein, we present an innovative microfluidic design that overcomes one of the current limitations of in vitro brain models: their inability to recapitulate the heterogeneity of brain regions in terms of cellular density and number. This device allows the controlled and uniform deposition of any cellular population within unique plating chambers of variable size and shape. Through the fine tuning of the hydrodynamic resistance and cell deposition rate, the number of neurons seeded in each plating chamber can be tailored from a thousand up to a million. By applying our design to so-called neurofluidic devices, we offer novel neuro-engineered microfluidic platforms that can be strategically used as organ-on-a-chip platforms for neuroscience research. These advances provide essential enhancements to in vitro platforms in the quest to provide structural architectures that support models for investigating human neurodegenerative diseases.

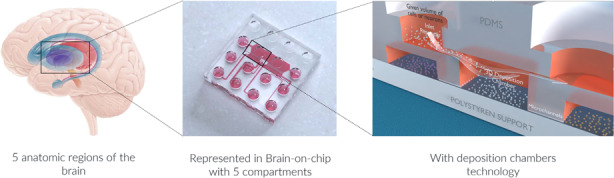

## Introduction

Members of the human population aged 60 years and older are increasingly affected by disorders of the central nervous system (CNS), such as Alzheimer and Parkinson diseases^1,2^. Even if massive research efforts have been made to find remedies for these disorders, effective cures have not yet been identified. For decades, the global use of mostly palliative therapies in patient health care has represented a significant cost for society (4.1% of EU GPID^1^). Despite the urgent need for appropriate medical treatments, pharmaceutical industries are unable to efficiently propose either curative or preventive measures^2^.

Progress in the diagnosis and treatment of neurological disorders is currently facing two bottlenecks. The first one can be addressed by fundamental research and concerns the extremely complex nature of the human brain^3^. The human brain includes hundreds of brain regions, a variety of different cell types^4^ and a connectivity pattern that has not yet been fully resolved. Such complexity makes it challenging to decipher a complete picture of the structure-function relationship that supports information processing within the brain and to find reliable biomarkers allowing the accurate evaluation of both the efficacy and the effectiveness of a specific therapy. The second bottleneck is linked to the nature of the currently used in vivo models for preclinical trials. Indeed, most in vivo models used to date for preclinical studies have neither structural nor functional translationality (*i.e*., the capacity of a research model to respond to a treatment in a manner similar to that of other models)^5^, leading to a low success rate of new therapies in clinical trials. Therefore, to overcome these challenges, there is an urgent need to find more relevant models for CNS research that can represent the complexity of the intact brain with higher fidelity than current animal or brain tissue studies and to act as bench-to-bedside technology.

Recent notable advances in in vitro modeling applied to neuroscience exploit the potential of organ-on-a-chip (OoC) microfluidic technology^6–8^ to regulate the connectivity^9–11^ and directionality^12–17^ between several compartmentalized neuronal populations and hence recreate simplistic, yet relevant, neuronal networks^9–14^. Neuro-engineered OoC microfluidics, also known as neurofluidic devices, demonstrate the capacity to isolate, control, and manipulate cellular environments^18,19^, allowing the coculture of different neural cell types while being fluidly isolated^15,20,21^. These approaches can be used as minimalistic in vitro models of in vivo neural circuits composed of connected nodes, which are clusters of specialized neuronal populations^12,22^. Although scientists have mainly focused on creating uni- or bidirectional connectivity patterns between nodes^6^, little work has been done on the inner architecture of the nodes themselves. Whether using microfluidic-based techniques^19,20,23,24^, colloidal support^25^ or scaffolded building blocks^26^, current state-of-the-art in vitro technologies still do not have control over the full architecture of the neural circuit with independent, while connected, organized nodes. We believe that this critical aspect is necessary to fully address the physiological relevance of brain-on-chip approaches. Thus, the next immediate step in the creation of OoC technology for CNS research is to standardize microfluidic strategies that can reconstruct complex but organized interconnected and compartmentalized neuronal networks, opening the path to studying their structure-function relationship.

In this study, we present a method for efficiently scaling and designing various neuro-engineered microfluidic devices to control the homogeneous seeding of neurons with a targeted number of cells within each individual node. Our results show the efficiency of this approach for a wide range of neuronal quantities while conserving uniformity in terms of the surface coverage of the plating chamber. Interfaced with existing strategies to control the connectivity between nodes, this technique has the potential to create multinode neurofluidic chips, holding structural connectivity patterns between several nodes. Such an exclusive ability allowed us to construct a microfluidic architecture for modeling the structural CNS circuits affected in neurodegenerative diseases, as the direct way of the basal ganglia loop in the brain is composed of five anatomical structures involved in Parkinson disease. If coupled to appropriate human-derived iPSC neurons in the future, such architecture would have the potential to create a minimalist yet relevant model of Parkinson disease.

## Results and discussion

### Deposition chamber concept and functionality of the system

#### Structural configuration of the design

Conventional microfluidic compartmentalization of neural cells uses a set of straight microchannels that connect two separated culture chambers^19,20,24,27^. Such a design requires the plating of a high quantity of neurons in small seeding volumes (~µL), and hence, a homogeneous neuronal density within the culture compartments of the device becomes difficult to achieve. Moreover, there is a significant loss of neuronal material because of the relatively small active areas (i.e., the opening sections of the microchannels), where growing neurites enter to reach the contiguous compartment, compared to the dimensions of the seeding compartment itself.

To resolve these issues, we propose the construction of supplemental compartments that fit the active areas to control the number and distribution of cells seeded. This design allows proper regulation of neuronal plating homogeneity across the culture chamber and therefore maximizes the connectivity pattern between compartmentalized neuronal populations. We have denoted such additional compartments as “Three Dimensional Deposition Chambers” (DCs).

**Fig. 1:**
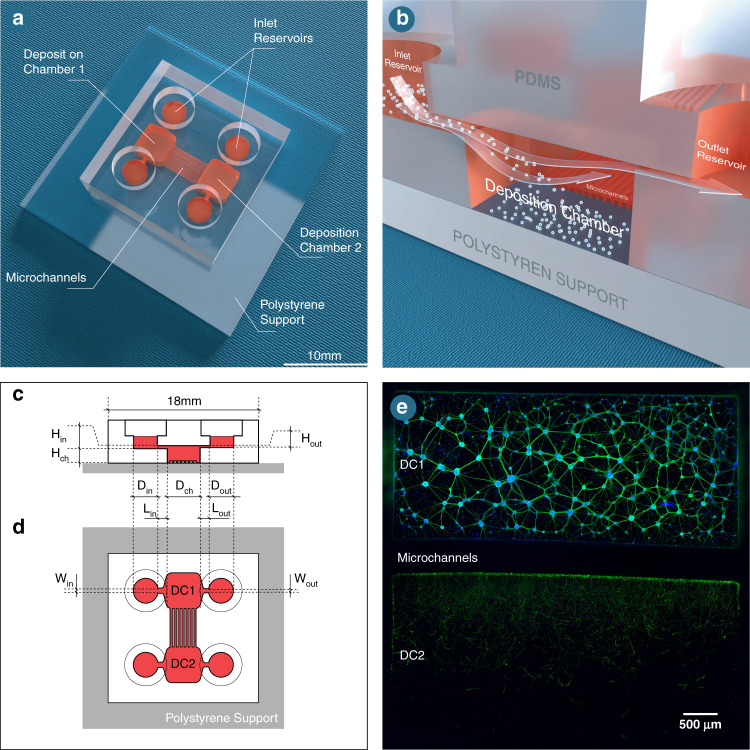
Overview of connected deposition chambers. **a** Schematic representation of a dual deposition chamber connected with microchannels. **b** Schematic representation of the deposition chamber (DC) technology. Illustrations of the **c** side and **d** top views of the model indicating which dimensions of the structural components are used to calibrate the flow profile within the culture compartments. **e** Immunofluorescent pictures of 18 DIV embryonic rat hippocampal with anti-BIII-Tubulin (Green) and with DAPI (bleu), seeded in a 10^5^ rectangular deposition chamber (DC1) connected via 450 μm long microchannels with another (DC2). Image was obtained using a x10 objective. The scale bar represents 500 µm

Since neurons seeded in the inlet and outlet plating channels of the device must not connect to those seeded within the DCs, we built them on an upper level in relation to the DCs (Fig. 1a, b). To exemplify our technology, two separated DCs are connected through a set of 450 µm long microchannels (Fig. 1c), which works as a barrier to keep cell bodies within the chamber while allowing neurites to pass through and extend into the connected chamber (Fig. 1d). To regulate the fluid velocity within each chamber, we designed channels that connect reservoirs to the DCs with distinct dimensions, with the inlet channel being higher and wider than the outlet channel (Fig. 1b, c). Such a feature of our design is the most important element that this innovative technology offers since the controlled flow velocity competes with the settling velocity of the neurons in the deposition chamber. Neurons that are closer to the top surface when entering the deposition chamber will reach the bottom of this chamber just before reaching the exit.

#### Flow velocity optimization

Based on the already mentioned structural configuration of our microfluidic design, none of the devices presented in this work require the use of pumping systems, in contrast to previously published microfluidic approaches^21,23,28–31^. As an alternative to such systems, we exploit the difference in hydrostatic pressure between the inlet and outlet reservoirs to generate a controlled flow. This flow is governed by the height difference of the free surface between both reservoirs and the hydrodynamic resistance throughout the channels and the DC. The hydrodynamic resistance is due to frictional forces acting against the motion of the fluid flowing through the channels, and such forces mainly depend on the geometrical properties of the various structural components. Hence, the precise dimensioning of these components is the central aspect of this technique, which is performed as detailed below.

First, the geometry and proportions of the DC are defined by taking into consideration the desired quantity of neurons to be seeded. The quantity of neurons that settle into the DC is correlated with the surface size of the chamber itself, meaning that the greater the surface, the more neurons can be deposited. As an example, a rectangular DC with a length (L_ch_) of 5000 µm, a width (W_ch_) of 2200 µm and a height (H_ch_) of 550 µm is presented in Fig. 1.

Second, the velocity of the suspension required in the DC (*V*_*ch*_) is evaluated according to the settling velocity of the neurons (*V*_*sedi*_). Indeed, if the flow velocity within the chamber is too high, the neurons in suspension will not have time to fall to the bottom of the chamber before being transported to the outlet channel; however, if the flow velocity in the chamber is too low, the neurons in suspension will reach the bottom too early, thus preventing their homogenous deposition. For a given dimension of the DC, the optimal flow velocity of the neuronal suspension is determined by:

1 $$V_{ch \,\,= }V_{sedi}\frac{{H_{ch}}}{{L_{ch}}}$$

**Fig. 2:**
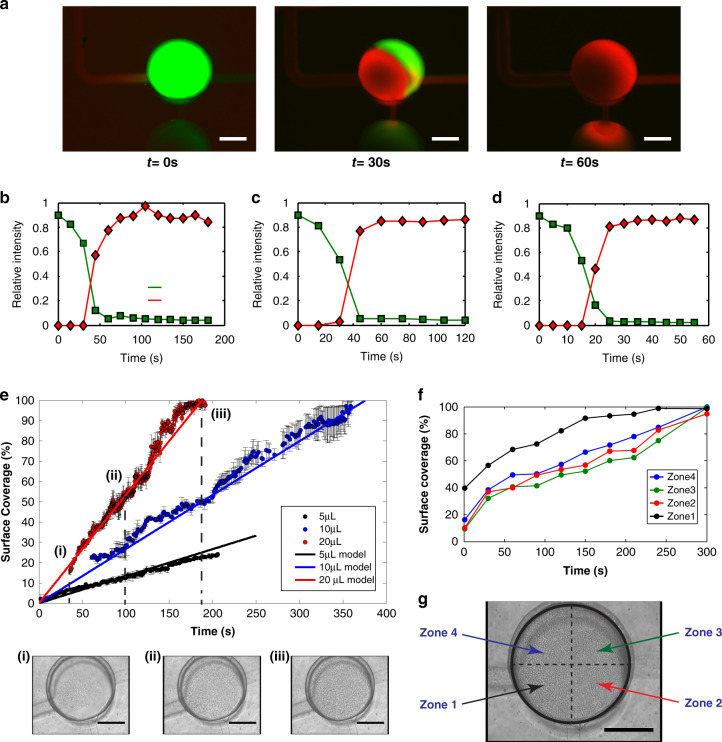
Flow measurments in a circular deposition chamber. **a** Assessment of fluid renewal within a cylindrical deposition chamber, where the exchange of fluorescein (green) and rhodamine 6 G (red) is analyzed at different time points (0, 30 and 60 s). Scale bars indicate 250 µm. Plots indicating the measured intensities of both fluorophores as a function of time (seconds) for **b** 4 µL, **c** 40 µL, and **d** 80 µL of inlet infused volumes of fluorescein (green) and rhodamine 6 G (red). **e** Quantification of the surface coverage of a cylindrical deposition chamber by neurons as a function of deposition time (seconds) for various inlet infused volumes. Solid lines indicate the model prediction for each condition, and discontinuous lines represent the surface coverage at *(*e.i*)* 35 s, *(*e.ii*)* 100 s, and *(*e.iii) 185 s after volume infusion. Transmission light microscopy images, and scale bars indicate 200 µm. **f** Quantification of the surface coverage of a cylindrical deposition chamber by neurons as a function of deposition time (seconds) based on the division of the chamber in four quarters, which are illustrated in panel **g**. Scale bar indicates 200 µm

It is worth noting that the DC does not have to be in the shape of a rectangle: if the DC were to be cylindrical (as in Fig. 2), the diameter of the chamber (*D*_*ch*_) would be used in Eq. (1) instead of *L*_*ch*_. The settling velocity of the neurons is estimated to be ~2µm/s by using the Stokes expression of the terminal velocity of a sphere falling in a fluid (estimating the diameter of a neuron in suspension to be approximately 6 µm), with the density of the sphere being 2% higher than that of the suspending fluid^32^.

Third, the hydrodynamic resistance in the device is calculated to reach the required flow velocity in the DC. To do so, two types of head losses must be considered:

a) The major head losses are pressure drops caused by viscous effects. At low Reynolds numbers, they can be estimated using the following equation:^33–35^

2 $$\Delta z = \frac{{\lambda \eta QL}}{{WH^3\rho g}}$$

Where *W*, *H* and *L* are the width, height and length of the channel, respectively; *Q*, *ρ* and *η* are the flow rate, density and viscosity of the fluid, respectively; and *Δz* is the difference in the height of the free surface of the fluid between the inlet and the outlet areas. The parameter *λ* is a friction coefficient, which can be approximated at low Reynolds numbers as:^33–35^

3 $$\lambda = 12\left( {1 - 6\left( {\frac{2}{\pi }} \right)^5\left( {\frac{H}{W}} \right)} \right)^{ - 1}$$

b) The minor head losses are linked to the geometrical alterations of the device (e.g., the modification of the cross-sections of the channels). Considering the range of flow rates used in this study, these head losses are considered of minor importance and thus are neglected during the dimensioning process of the microfluidic device. Therefore, the only unknown parameters are the widths, heights, and lengths of both the inlet and outlet channels.

In this work, we fixed the widths and the heights of these channels at values that are optimized for easy operation of the system. In the device depicted in Fig. 1e, for example, the inlet channel has a width (*W*_*in*_) of 1000 µm and a height (*H*_*in*_) of 100 µm that were selected to avoid any possible clogging, whereas the outlet channel has a width (*W*_*out*_) of 1000 µm and a height (*H*_*out*_) of 50 µm. The inlet channel length (*L*_*in*_) was set to 1000 µm to reduce neuronal settling within it. Finally, we optimized the length of the outlet channel (*L*_*out*_) to ~9900 µm to obtain the required flow velocity in the DC using the previously defined equations.

Once all the previous parameters are estimated, the geometries and positions of both the inlet and outlet reservoirs, as well as the infused volumes of cell suspension, need to be determined. In our presented design, both inlet and outlet reservoirs are cylinders with respective diameters (*D*_*in*_ and *D*_*out*_) of 4 mm (Fig. 1b, c), and the infused volume of cell suspension was 20 µL.

#### Model of the flow profile and deposition rate of cells

To precisely monitor the speed of the flow within our neuro-engineered microfluidic devices, we tested the design and utilization parameters (such as pressure difference and cell concentration in the suspension) before the fabrication step by developing a simplistic model of the neuronal deposition process.

Considering the inherent complexity of this multiphase process, we intended to simplify the modeling procedure by using three assumptions: i) The flow is perfectly laminar everywhere within the microfluidic chip (as the calculated Reynolds number is inferior to 1); thus, the flow profile can be simplified as a perfect Poiseuille flow all along the device. ii) The velocity of the flow depends on the difference in hydrostatic pressure between the inlet and the outlet areas, the hydraulic resistance, and the viscosity of the fluid, which is considered as a Newtonian fluid. iii) The layer of cells covering the bottom of the DC is perfectly unicellular and arranged in a regular hexagonal close packing configuration at maximum packing^36^. Therefore, the neuronal coverage of the bottom surface of the DC (*ϕ*) can be defined as:

4 $$\phi = \frac{{N \times \pi a^2}}{S}\frac{\pi }{{3\sqrt {2}}}$$

where *N* is the number of neurons in the DC, *a* is the radius of a neuron, and *S* is the bottom surface size of the chamber. As a result, the neuronal coverage of the surface is a dimensionless value, varying between 0 (if no neurons are attached to the surface) and 1 (if the surface is fully covered with neurons). The evolution of this value as a function of time is governed by the evolution of *N* as a function of time, which can be expressed as the product between the neuron concentration in the suspension (*φ*) and the volume of the suspension that entered the DC (*V*):

5 $$N = \varphi \times V$$

As *φ is* the neuron concentration already measured and corrected at the beginning of the experiment, the only unknown value is the volume of the suspension, which can be calculated as the product of the flow rate in the device (*Q*) and the experimental time (*t*):

6 $$V = Q \times t$$

The flow rate is defined as the product between the mean velocity of the fluid in the deposition chamber and the cross-section of the chamber and can be fully evaluated in the device. The flow specifically depends on the difference in hydrostatic pressure between the inlet and outlet channels and on the hydrodynamic resistance in the device. If the defined experimental time is short, the hydrostatic pressure difference does not change significantly; thus, it is not necessary to discretize in time. Otherwise, a straightforward time discretization allows us to consider the fluid volume difference occurring between the inlet and the outlet areas, therefore modifying the change in hydrostatic pressure and the flow rate.

To ensure that the calculations are in accordance with the actual flow in the device, the velocity of the fluid was measured in both the inlet and outlet channels using fluorescent particles with a diameter of 1 µm. Three volumes were tested in the inlet channel, and for each volume, time-lapse images of both the inlet and outlet channels were obtained. Knowing the geometrical properties of the channels and the inlet volume infused, it is possible to calculate the theoretical Poiseuille profile of the flow velocity within the channels. We found that inlet volumes of 20 µL, 40 µL and 60 µL show maximum flow velocities of 43 µm/s, 90 µm/s and 130 µm/s within the inlet channel and 160 µm/s, 315 µm/s and 450 µm/s within the outlet channel, respectively (data not shown). These measurements are found to be in good agreement with the theoretical velocity profiles (Fig. S1a).

To achieve fine control of the fluid flow relative to the rate at which the neurons cover the surface, defining the speed of fluid or media renewal within the chamber is crucial. To do so, we analyzed the time needed for rhodamine to substitute fluorescein within the DC (Fig. 2a). For several infused volumes of fluid, we obtained a direct correlation between the infused volume in the inlet reservoir and fluorophore exchange within the chamber (Fig. 2b–d). Once the fluid renewal speed was determined, the deposition rate could be adjusted.

To optimize this feature, three different volumes of neuronal suspension (5 µL, 10 µL and 20 µL) were added to the inlet channel at a concentration of 5 × 10^7^cells/mL. The flow can be stopped at any time by equalizing the volume between the inlet and the outlet areas. Thus, the deposition process can be interrupted by either removing the fluid in these areas or by adding a corresponding fluid volume at the outlet area. We showed that with an inlet volume of 20 µL at 5 × 10^7^ cells/mL, the surface of the DC was completely covered by neurons after 185 s (Fig. 2e).

#### Neuron density and uniformity

As previously mentioned, the homogeneous distribution of cells seeded within microfluidic devices is still a challenge to overcome. In our system, the uniform allocation of the deposited cells was verified by splitting the deposition surface into four quarters and monitoring the cell density over time during the deposition process. At the end of this process, seeded neurons covered most of all four quarters of the deposition surface (Fig. 2f). Importantly, neurons covered the surface of zone 1 20% faster than those of other zones due to its position in relation to the inlet channel. Nevertheless, the layer was uniform by the end of the deposition process (Fig. 2g).

#### Geometry variability

**Fig. 3:**
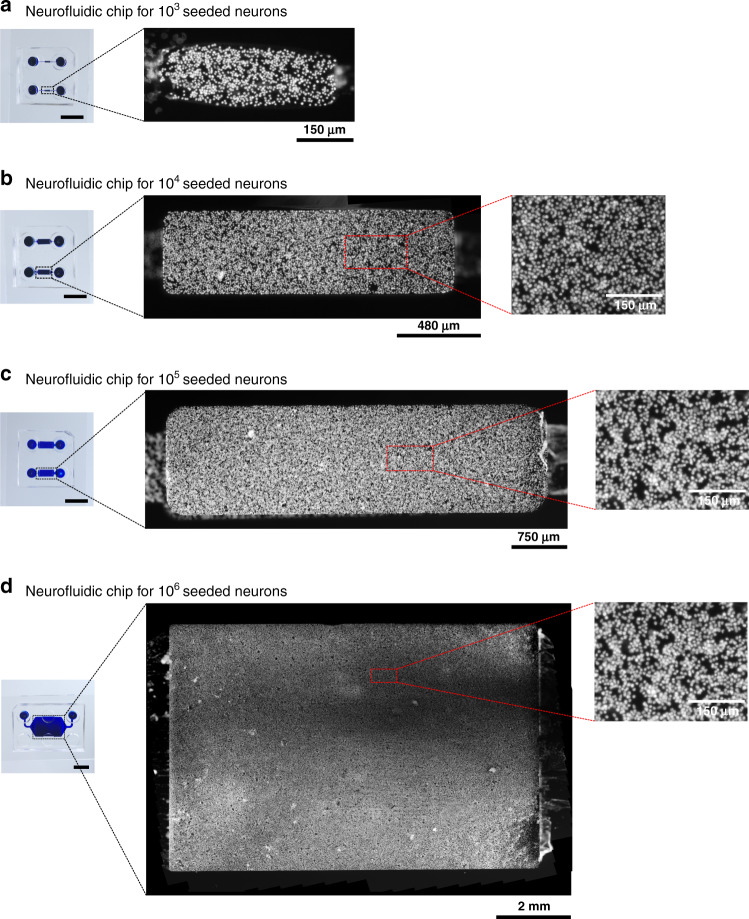
Illustrative pictures of DAPI-stained neurons seeded within rectangular deposition chambers. **a** 10^3^ neurons, **b** 10^4^ neurons, **c** 10^5^ neurons, and **d** 10^6^ neurons one day post-seeding. The scale bars illustrated on the images of the chips indicate 10 mm

The geometry of the microfluidic device can be modified to suit neuronal populations of any size. Four conditions were specified to target specific quantities of neurons: 10^3^, 10^4^, 10^5^ and 10^6^ neurons. For clarity, these devices were referred to as N1e3, N1e4, N1e5 and N1e6 devices, respectively (Fig. 3). To obtain controlled cell deposition distributions in the chambers, both the inlet and outlet channels of the device were scaled using the previously described dimensioning method. All four devices were filled with primary rat hippocampal neurons, and cell nuclei were stained with DAPI after deposition to perform cell counting in the microfluidic chambers. We detected that the desired number of deposited neurons in each device was reached at 95% (Fig. S1b). The missing 5% of surface coverage might be due to an error in terms of deposition timing (as one second too late or too early when stopping the flow induces a 5% error in terms of surface coverage).

To better assess the uniformity of the cell distributions after deposition along the direction of the flow, the acquired images of the DCs from all devices were divided into ten rectangular sections, perpendicular to the flow, from near the inlet channel (Section n°1) until near the outlet channel (Section n°10). Then, the surface coverage by neurons was measured within each section. The results showed that the neuronal distributions along the surfaces of the DCs were conserved throughout all devices (Fig. S1c). To further verify the overall homogeneity of the seeding, each DC was presented as a matrix of squares representing the quantification of surface coverage compared to regular microfluidic channels (Fig. S1d). These results confirmed the overall homogeneity of the seeding for three independent seeding experiments with different operators.

#### Neuronal seeding and viability

**Fig. 4:**
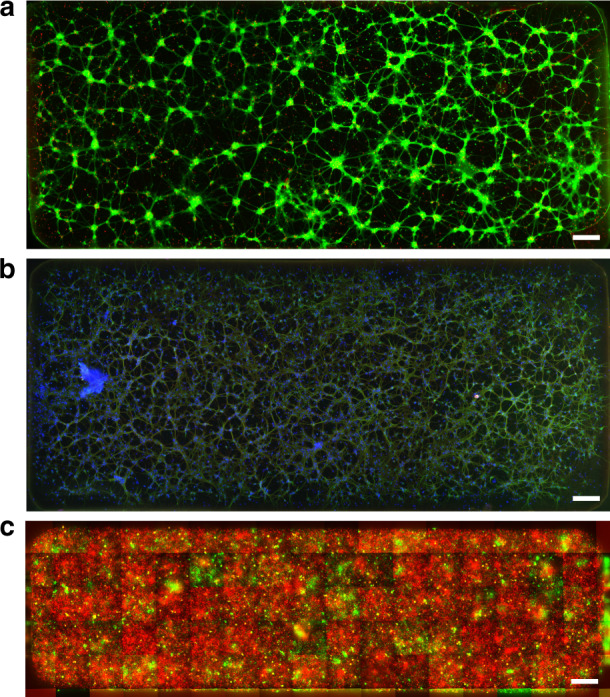
Illustrative pictures of embryonic rat hippocampal cell culture at 18 DIV. **a** Staining with the LIVE/DEAD® Viability/Cytotoxicity Kit for the assessment of alive cells (green) and dead cells (red). **b** Visualization of axons Tau (red) and dendrites MAP2 (green) against and counterstained with DAPI (blue). **c** A population of hippocampal neurons was split in two halves and stained using the Vybrant^™^ Multicolor Cell-Labeling Kit for live-cell imaging, incubating one half with DiO solution (green), and the other half with DiD Solution (red). The mosaic of fluorescent images shows an 80% surface coverage of red cells, and a 20% coverage of green cells. All images were obtained using a x10 objective. All scale bars indicate 200 µm

Knowing that the uniformity of cell distributions within the DCs after seeding was successfully attained, we questioned whether the adhered neurons showed healthy morphologies. Such features were confirmed using 18 days in vitro (DIV) neurons seeded into the DC of an N1e5 device, which showed up to 80% cell viability (Fig. 4a) and homogeneous interconnectivity while immunostaining against MAP2 and Tau to visualize neurites (dendrites and axons) (Fig. 4b).

Furthermore, we aimed to demonstrate that sequential seeding could be performed in DCs. Using a live cell-staining reagent, one population of neurons was labeled with green fluorescence, while the other population was labeled with red fluorescence before seeding. We thus seeded the red-labeled cells first, aiming at 80% coverage, followed by the green-labeled population, aiming at 20% coverage, in an N1e5 device. The results showed that we could cover ~80% of the surface of the DC with the red population while covering ~20% with the green population (Fig. 4c), showing the capability of the DC to coculture several cell types within the same chamber with given cell population proportions. Although this could be achieved while seeding premixed populations at the target proportions, DCs ensure the maturation of cells over time while accepting, in a homogenous way, another cell type to be seeded at a separate seed time. Such a situation could be necessary when cell types do not require the same maturation time, for example, with human induced pluripotent stem cell-derived (iPSC) neurons and glial cells, or to monitor the influence of one cell type on mature neural cells, for example, adding cancer cells to mature neural cells.

#### Control of the surface coverage of the DC

As previously described, the complete coverage of the DCs in the four presented devices was attained thanks to the infusion of a 20 µL neuron suspension with a concentration of 5 × 10^7^cells/mL, and consequently, the flow was stopped. Nevertheless, if needed, the flow can also be interrupted at any time during the process when partially filling the DC, although the homogeneous distribution of neurons is not guaranteed (data not shown). Another approach to achieve this goal is to take advantage of the linearity between the number of neurons entering the chamber and the concentration of the cell suspension. As the time required to entirely cover the bottom surface was verified, the neuron concentration within the suspension could be changed and used with the same deposition time. For example, an N1e6 device was filled with 20 µL of neuron suspension at a concentration of 1.25 × 10^7^cells/mL (1/4 of the amount used to completely cover the chamber’s surface) without modifying the deposition time (20 s). As expected, only 20% of the surface was then covered during seeding, with the same homogeneity qualities along the DC (Fig. S2). Hence, controlling the neuronal concentration in suspension enabled partial, yet homogeneous, neuronal seeding in the device.

### Translation of in vivo neural circuits to in vitro neurofluidic architectures

Currently used OoC technologies applied in neuroscience research have mostly focused on the creation of aligned and linear cell-cell interfaces, ranging from 2^15,37^, 3^38^ or 5^39^ connected compartments, rather than fabricating an in vitro model that specifically mimics the complex 3D connectivity across various brain regions. The brain is composed of thousands of neural cell types entangled in a highly intricate and structured network, forming circuits between several substructures that support information processing. Hence, such complexity must be considered when developing novel neuro-engineered OoC systems. Several attempts have been made to increase connectivity relevance^10^, albeit without the capacity to scale nodes or increase connectivity patterns. We believe that DC technology has the potential to overcome such limits. By using all the borders of the chamber itself, nodes can be connected to others using directional or bidirectional channels in all directions, allowing a complex connectivity architecture.

We believe that OoC technology needs to present a high degree of standardization to be fully accepted by the industry as reliable platforms in preclinical drug screening assays. To date, most pharmaceutical and biotechnology companies use the SBS (“Society for Biomolecular Screening”) ANSI (“American National Standards Institute”) format, either in automated liquid handling robots to perform cell culture or in high content screening platforms for immunoassay readouts. We believe that all OoC technologies should follow such a design to boost acceptance.

For brain and neuroscience applications, brain-on-chip technology requires both physiologically relevant architectures, which connect tens to millions of human neurons with complex connectivities, and electrophysiological recordings at the entire network level. To date, only a multielectrode array (MEA) of high density multielectrode arrays (hdMEA) has the capacity to capture the spatiotemporal connectivity of a neural network. To exemplify the compatibility of DC technology and MEAs, we seeded neurons on a DC and recorded signals for ten minutes: neuronal spikes were perfectly identified, showing maturity and functionality of the network (Fig. S3).

**Fig. 5:**
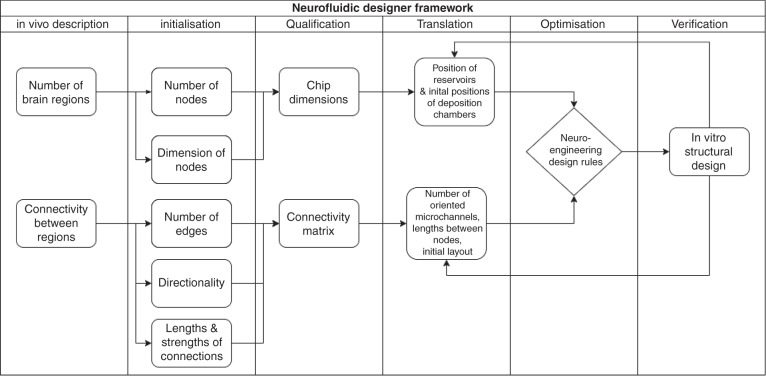
Schematic overview of the neurofluidic designer framework for the transformation of any in vivo brain circuit to its in vitro neuro-engineered microfluidic architecture, including structural (deposition chambers and connections) and functional (MEA) aspects to be determined on the chip

To facilitate such brain-on-chip in vitro model development, we present a specific translational construction framework to accurately translate in vivo neural circuits into in vitro neurofluidic architectures coupled to MEAs (Fig. 5). This framework implements the DC technology presented herein while maintaining the alignment of the reservoir in the SBS ANSI format due to the versatility in the design process of the inlet and outlet channel parameters.

The framework was separated into three main steps: (i) the first step included the characterization of the in vivo circuit of study, where the elements composing the circuit were deciphered into quantifiable factors (e.g., the number of nodes per brain region involved, the dimensions of the individual nodes, etc.). This first step required establishing a formal description of the in vitro neural network, which was performed using the JSON descriptor (example given in File S4). (ii) The second step involved the design of an initial layout of the neurofluidic device based on these factors (e.g., position of the DC, number and directionality of microchannels, etc.). (iii) The final step was used for the optimization and verification of the designed device, where the obtained feedback could be applied to the second step for optimization.

To familiarize the community to such an exchange format using JSON descriptors, the framework has been implemented on open-source software that can be found on http://designer.netri.fr/ (source code on demand). Importantly, those files can be shared on open microfluidic platforms to be used for free by the scientific community for the design of their own neurofluidic chip of interest.

### Basal ganglia circuit on-a-chip using the deposition chamber system

By applying the previously mentioned framework, we reported the use of deposition chamber technology for the construction of a five-nodal microfluidic chip to provide an improved in vitro model of the basal ganglia loop direct pathway, whose functionality is known to be affected in Parkinson’s and Huntington’s diseases^40^.

**Fig. 6:**
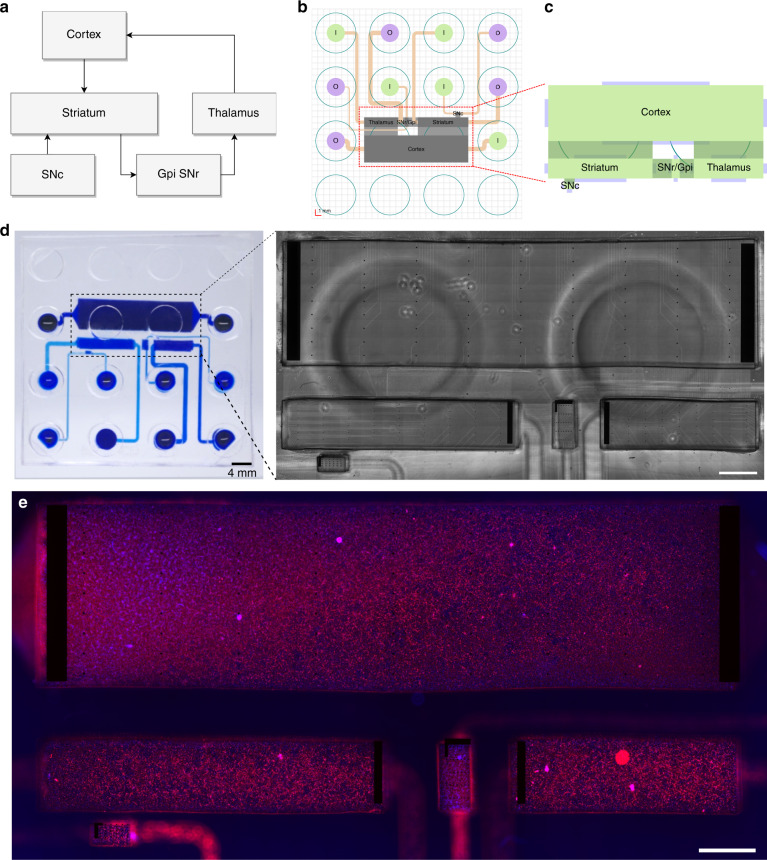
Implementation of the neurofluidic framework for the construction of the basal ganglia circuit on a chip. **a** Scheme representing the regions and connections within the in vivo circuit. GPi: Glubulus Pallidus internal, SNr: Substantia Nigra reticularis SNc: Substantia Nigra compacta. **b** Structural setting and positioning of the inlet and outlet channels, together with their respective input and output reservoirs. **c** Schematic representation of the in vitro application of the deposition chambers. **d** Image of the reconstructed basal ganglia circuit on a chip using the deposition chamber technology, where all compartments are filled with blue ink. Inset: Transmission light microscope image of the multi-electrode array aligned on the neurofluidic architecture. The image was obtained using a x10 objective. **e** Immunofluorescent pictures of 18 DIV embryonic rat hippocampal with anti-MAP2 (Red) and with DAPI (blue). All images were obtained using a x10 objective. Scale bars represent 1 mm

Neural circuits of the CNS are established by convoluted neuronal networks linked by extensive axonal processes. The basal ganglia loop consists of separated nodes positioned in different areas of the deep brain. Consequently, we first aimed to recreate the structural architecture of the in vivo basal ganglia loop on a conventional PDMS chip using different subtypes of neurons obtained from rat embryos (Fig. 6). Based on a substantial analysis of a 3D model of the rat brain^41^, we extracted the specific number of neurons required for the reconstruction of each node from the basal ganglia loop direct pathway. The seeding spectrum ranged from ~2 × 10^3^ up to ~5 × 10^6^ neurons within all DCs of the future device, depending on the reconstructed region (Table 1). The adequate positioning of the nodes and the control of their inherent connectivity are crucial to precisely mimic the in vivo structural association among these regions on the device. Therefore, nodes were aligned on the chip with respect to the converted required surface and subsequently linked using several arrays of microchannels (Fig. 6b, c).

**Table 1:** Absolute volumes and rounded matching numbers of neurons in the major nodes constituting the cortico-basal ganglia-thalamo-cortical loop of the rat brain^41^

	Absolute volumes (mm^3^)	Number of neurons
Cortex	470	5 000 000
Thalamus	48	510 638
Striatum	72	765 957
GPi/SNr	6	63 830
SN	4,3	45 426

We integrated our DC technology into the optimized in vitro model of the basal ganglia loop. To respect the connectivity pattern between the inlet/outlet channels and their respective reservoirs, the inlet and outlet reservoirs for each node were positioned onto the chip by considering the design rules previously described (Fig. 6b). Since cultured neurons extend their projections from node to node via the connecting microchannels, functional assessment is essential to confirm the correct electrical communication among nodes. In accordance, the activity of these networks can be validated by coupling the neurofluidic chips with electrophysiological recording systems (Fig. 6d) that have been specifically designed to fit an even distribution throughout the nodes in a 256 electrode MEA. For example, we seeded rat hippocampal neurons within the DC of the basal ganglia loop device (BG5 device), wherein (MEA) were incorporated. The full characterization of the BG5 device using dedicated human neuronal types in each node falls beyond the scope of this work and will be explored further to model Parkinson disease on-chip devices.

## Conclusions and perspectives

OoC technologies are state-of-the-art research tools that allow the construction of in vitro models with an accurate structural design at the organ level and hence can be used to identify potential molecular and cellular factors in human pathophysiology. This study describes an innovative microfluidic approach for creating improved neuro-engineered OoC devices via the control of neurons seeded into deposition chambers for the reconstruction of minimalistic brain connectomes. We applied this system to build an in vitro multinodal depiction of the basal ganglia circuit of the brain, whose dysfunction leads to neurodegeneration in Parkinson disease.

Herein, we demonstrated the efficiency of this pumpless neurofluidic technology to homogeneously plate neuronal populations at controlled densities into microfluidic culture chambers, enabling the fabrication of simplified brain networks formed by realistic proportions of various neuronal subtypes. This promising method, in combination with a multinodal patterning approach, represents a major advancement toward modeling the complex neural circuitry present in the intact brain by replicating functional and reliable connectomes-on-a-chip. Moreover, this work introduces a new technological design framework to engineer on a chip any existing neural circuit of interest for disease pathways currently under study and to provide the scientific community with standards matching industrial applications, allowing a faster standardization and adoption of OoC technology by the pharmaceutical industry.

There are still challenges remaining for the validation of the OoC basal ganglia loop complete model, including accurate neural subtypes seeding in each node, controlled directional connectivity between nodes and network-wide electrophysiological recordings and connectivity mapping. Future work should focus on recording the local application of alpha synuclein in the SN compartment and monitoring the effects of typical pharmacological standards to offer a physiologically relevant and predictive model.

## Materials and methods

### Masks and SU-8 mold fabrication

For the construction of all two-layered compartmentalized chips, respective masks for the designed layers were purchased on transparent films (Selba SA, CH). The molds required to fabricate the microfluidic devices were made using conventional photolithography techniques and an SU8 photoresist series (MicroChem, USA).

### PDMS microfluidic chip fabrication

The wafers for the top layers (patterned with the inlet and outlet channels) were silanized using a silanizing agent (trichloro(1H,1H,2H,2H-perfluorooctyl)silane) in a desiccator for 30 min. Polymethylsiloxane (PDMS) prepolymer (Sylgard 184, Dow Corning, USA) was prepared and cast onto the molds before being cured in an oven at 80 °C for 40 min. Subsequently, the PDMS layers were cut to the required size before being peeled off the molds. The inlet and outlet zones were then punched out, and the PDMS was cleaned and protected using adhesive tape.

For the bottom layers of the devices, the molds were cut to the required size and placed onto individual microscope glass slides. PDMS prepolymer was then cast onto them. Subsequently, a 2 mm thick film of polyimide (CS Hyde, USA), stacked on paired microscope glass slides, was placed onto the PDMS while carefully applying a slight pressure. The stacks were then cured in an oven at 80 °C for 40 min. Once cured, the upper microscope glass slides were gently removed, and both the polyimide sheet and the thin PDMS layer were cut to the desired size and removed from the molds. The resulting layers and clean microscope glass slides were then plasma-treated using a plasma cleaner (Harrick Plasma, USA) before being assembled. Later, the polyimide sheet was gently removed, and both PDMS top and bottom layers were plasma-treated again before their assembly. The constructed devices were finally sprayed and filled with a solution of 70% ethanol and brought into a sterile environment.

### Microfluidic device sterilization and functionalization

The 70% ethanol within the microfluidic devices was washed away three consecutive times using sterile distilled water, and then the devices were exposed to UV light for 30 min. The plating channels and the deposition chambers of the microfluidic devices were then coated using 0.1 mg/mL poly-L-lysine (Sigma Aldrich, USA) and placed in an incubator. After 24 h, the coated surfaces were rinsed three times with Hank’s Balanced Salt Solution (HBSS) (Life Technology, Thermo Fisher Scientific Inc., USA) buffered with 10 mM 4-(2-hydroxyethyl)−1-piperazineethanesulfonic acid (HEPES) (Life Technology, Thermo Fisher Scientific Inc., USA) and coated with 20 μg/mL laminin (Sigma Aldrich, USA) for 2 h. The coated devices were washed again three times with HBSS and then filled with neuronal culture medium composed of Neurobasal-B27 (Life Technology, Thermo Fisher Scientific Inc., USA) containing 2 mM glutamine and 100 U/mL penicillin/streptomycin (Life Technology, Thermo Fisher Scientific Inc., USA). The microfluidic chips were finally placed in an incubator until use.

### Neuron preparation and culture

All animal work was approved by the CEA and CNRS Ethics Committee of Animal Care and abided by institutional and national guidelines for animal welfare. Experiments performed at NETRI were approved by regional authorities for animal welfare (DDHS Agreement SPA-2019-19).

Neurons were harvested from E18 OFA rats (Charles River Laboratories) and kept in ice-cold HBSS buffered with 10 mM HEPES (pH 7.3). The tissue was digested for 30 min using 2 mL of HEPES-buffered HBSS containing 20 U/ml papain (Worthington Biochem., USA), 1 mM EDTA (PanReac AppliChem) and 1 mM L-cysteine (Sigma Aldrich, USA). Then, the tissue was rinsed three times with 8 mL of neuronal culture medium. The cells were gently triturated in 1 mL of neuronal culture medium, counted with a microfluidic cell counter (Scepter 2.0, Merck Millipore), and flowed into the devices. The cells were maintained under incubation conditions (37 °C, 5% CO_2_, and 80% humidity) until use.

Before seeding, the inlet/outlet reservoirs of the microfluidic chips were emptied without removing the media from the channels. Unless stated otherwise, 20 μL of high density (~5 × 10^7^cells/mL) dissociated neuron solution was placed near the entrance of the channels. The chips were returned to the incubator for 15 min to allow the neurons to adhere to the coated surfaces, and then both inlet and outlet reservoirs were filled with medium. While neurons were maintained in culture, the feeding medium was renewed at least three times per week. To do so, the inlets and outlets were emptied before adding 100 µL of fresh media to the inlets.

### Immunocytochemistry

For cell body visualization, 21 DIV neurons were fixed with a 4% paraformaldehyde (PFA) (Sigma–Aldrich) solution in PBS (Sigma–Aldrich) for 20 min. The cells were rinsed five times with PBS and subsequently permeabilized and blocked with a 3% bovine serum albumin (BSA) (Sigma–Aldrich) and 0.1% Triton X-100 (Sigma–Aldrich) solution in PBS for 45 min. Neuronal bodies and projections were labeled with antibodies against beta-3 tubulin (mouse monoclonal, Thermo Fisher, MA1-118, dilution 1:200; secondary antibody: goat anti-mouse IgG (H + L) Alexa Fluor 488, Life Technologies, dilution 1:1000), MAP2 (rabbit polyclonal, Thermo Fisher, PA5-17646, dilution 1:1000; secondary antibody: goat anti-rabbit Alexa Fluor 488, Life Technologies, dilution 1:1000) and Tau (mouse monoclonal, Thermo Fisher, AHB0042, dilution 1:1000; secondary antibody: goat anti-mouse Alexa Fluor 647, Life Technologies, dilution 1:1000). Image acquisition was performed with an AxiObserver A1 Microscope (Zeiss, Germany) using 10x and 20x objectives.

### Cell number quantification

To quantify the number of cells present within the chambers, 2 DIV neurons were fixed and permeabilized as described previously. The neurons were subsequently washed five times with PBS and incubated with a 300 nM DAPI solution (Thermo Fisher) for 10 min. After incubation, the cells were washed five times with PBS. Image acquisition was performed with an AxiObserver A1 Microscope (Zeiss, Germany) using 10x and 20x objectives.

### Live-cell imaging

For the optimization of the seeding steps for the devices, before seeding, cells were split into two halves and stained independently using cell membrane fluorescent markers from the Vybrant™ Multicolor Cell-Labeling Kit (V22889, Thermo Fisher Scientific Inc., USA). One half was incubated with 5 μM DiO Labeling Solution (green), and the other half was incubated with 5 μM DiD Labeling Solution (red). These incubations were performed on cells in suspension for 15 min at room temperature or 37 °C depending on the supplier’s recommendation. Then, the neurons were washed three times through centrifugation and resuspended in neuronal culture medium. Neurons were finally injected into the microfluidic devices before imaging.

Cell viability assessment was performed using the LIVE/DEAD™ Viability/Cytotoxicity Kit (L3224, Thermo Fisher Scientific Inc., USA), incubating neurons on culture with a Calcein AM-Ethidium solution for 30 min at room temperature. Cells were imaged without removing the staining solution.

### Image analysis

The obtained microscope images were stitched using the stitching plugin from ImageJ (NIH) to reconstruct the entire device. Later, every single reconstructed image was split into smaller images (to a hundredth the size of the original) with a self-developed macro in ImageJ. The brightness and contrast of each small image were adjusted before thresholding, and the surface coverage of the nuclei was then quantified. Only those images containing clearly separated DAPI-stained nuclei were selected for quantification. To extract an average nuclear size value, the size of each individual nucleus was measured. This resulting average value was then used to measure the device surface covered by nuclei to finally estimate the number of seeded neurons inside the deposition chambers.

Homogeneity evaluation was performed by splitting each obtained image into ten sections, and neurons were counted within each section. A ratio was calculated by dividing the measured number and the expected number of neurons on each section.

### Measurements of flow velocity in the devices

To measure the velocity of the fluid within the devices, three different volumes (20 µL, 40 µL, and 60 µL) of a suspension of 1 µm diameter fluorescent particles (FluoroMax, Thermo Fisher Scientific Inc., USA) at a concentration of 2 × 10^7^particles/mL was added to the inlet reservoirs. Dilutions were performed in neuronal culture media. The flow was then recorded using an AxiObserver Z1 microscope (Zeiss, Germany). Finally, the particles were manually tracked using ImageJ (NIH).

### Renewal of fluid within the deposition chambers

The devices were filled with a solution of fluorescein sodium (Sigma Aldrich, USA) diluted in distilled water (1 mg/mL). Then, various volumes of a solution of rhodamine 6 G (Sigma Aldrich, USA) diluted in distilled water (1 mg/mL) were added to the inlet channels, and the devices were imaged at various time points using an AxiObserver Z1 microscope (Zeiss, Germany).

## Supplementary information

SI description

SI Figure 1

SI Figure 2

SI Figure 3

SI Figure 4

SI Figure 5
