# Supplementary material for: Deposition chamber technology as building blocks for a standardized brain-on-chip framework
Source: Microsyst Nanoeng. 2022 Aug 1;8:86. doi: 10.1038/s41378-022-00406-x (PMC9339542; doi:10.1038/s41378-022-00406-x)
Supplement: Supplementary file 1 — SI description [file 41378_2022_406_MOESM1_ESM.docx]

Supplementary information

**Figure S1:** Neuronal seeding accurate with the deposition chamber technology: ***(a)*** graph plotting the number of neurons per mm² according to the cellular concentration in suspension before seeding in a deposition chamber from a N1e5 device. ***(b)*** Ratio between the estimated and the expected number of neurons in the deposition chamber devices. ***(c)*** Uniformity evaluation of seeded neurons within each deposition chamber device. ***(d)*** Heat map pictures showing seeding homogeneity in the different deposition chamber.

**Figure S2:** Seeding adjustment within deposition chambers: ***(a)*** Neurons were stained with DAPI within a partially filled deposition chamber of a N1e6 device. The final neuron count within the chamber was ~2.4x10^6^. The image was obtained using a 10x objective. ***(b)*** Uniformity evaluation of the surface coverage across the partially filled deposition chamber (20 %).

**Figure S3:** Introduction of electrophysiological recording systems into the devices: ***(a)*** Transmission light microscopy image of 21 DIV rat hippocampal neurons seeded in a N1e5 deposition chamber coupled to a multi-electrode array. A punch hole was performed prior to perform recording to insert Ag counter electrode on the lower left side of the image. The image was obtained using a 10x objective. Scale bar indicates 200 µm. ***(b)*** Example of the electrophysiological recording on one electrode. ***(c)*** Raster plot following spike analysis of the 10 minutes recorded signals on all electrodes.

**File S4:** JSON description of the basal ganglia circuit of the brain direct way on chip.

**Figure S5:** Independent pictures of merge picture presented in Figure 2-a. Picture represent fluorescein (green) and rhodamine 6G (red) at different time points (0, 30 and 60 seconds). Scale bars indicate 150 µm.
