# Supplementary figures and images for: Deposition chamber technology as building blocks for a standardized brain-on-chip framework
Source: Microsyst Nanoeng. 2022 Aug 1;8:86. doi: 10.1038/s41378-022-00406-x (PMC9339542; doi:10.1038/s41378-022-00406-x)

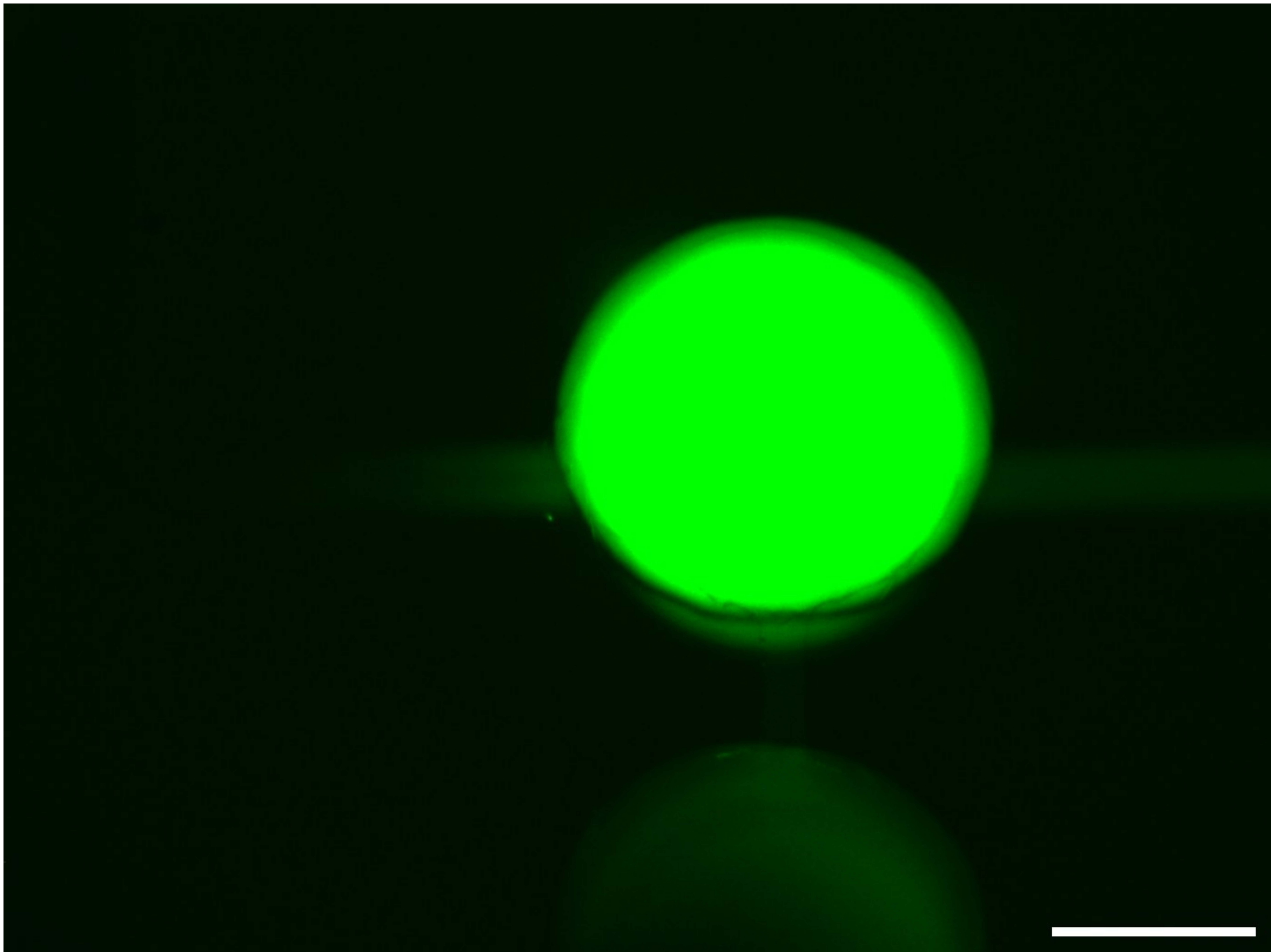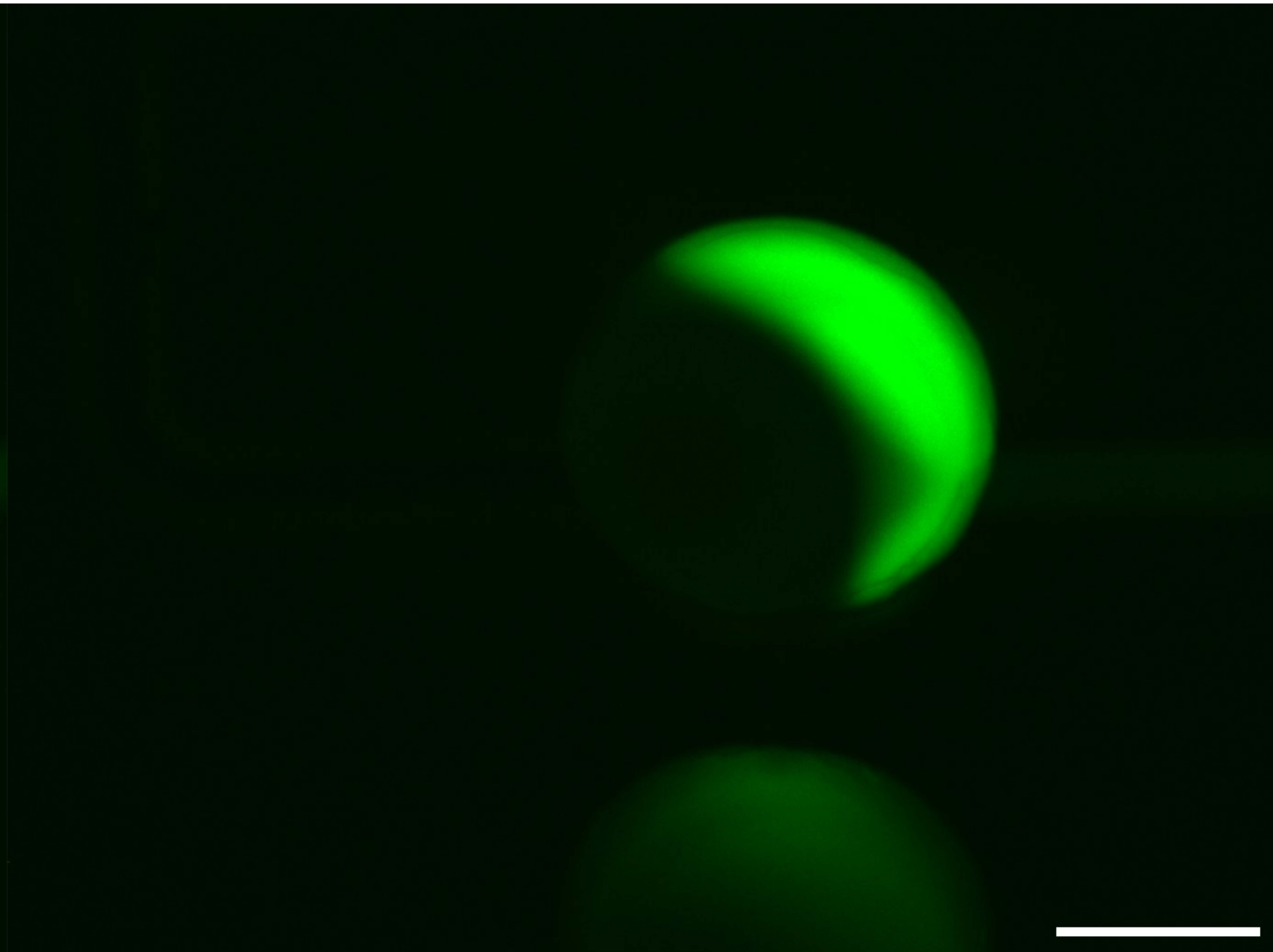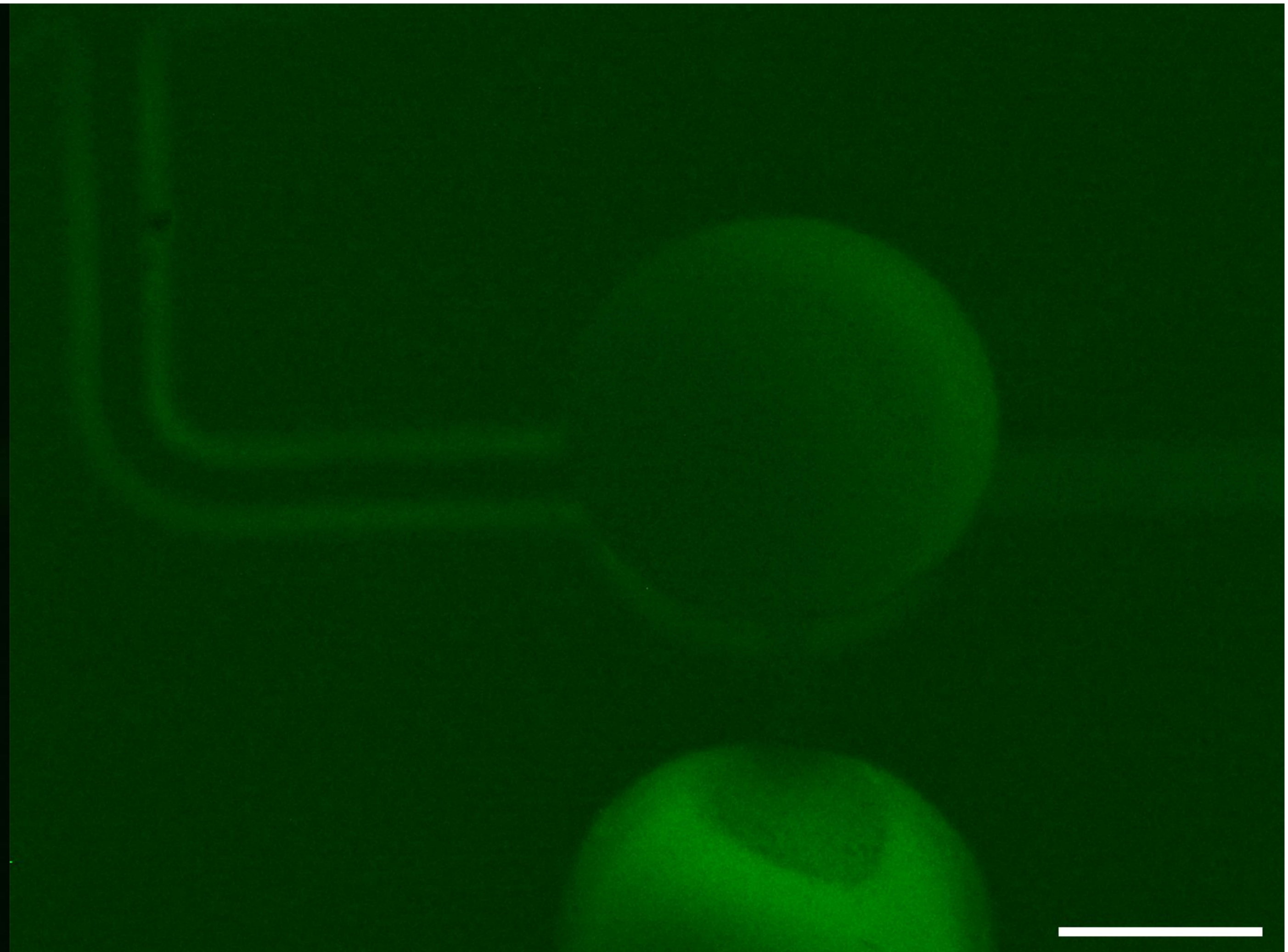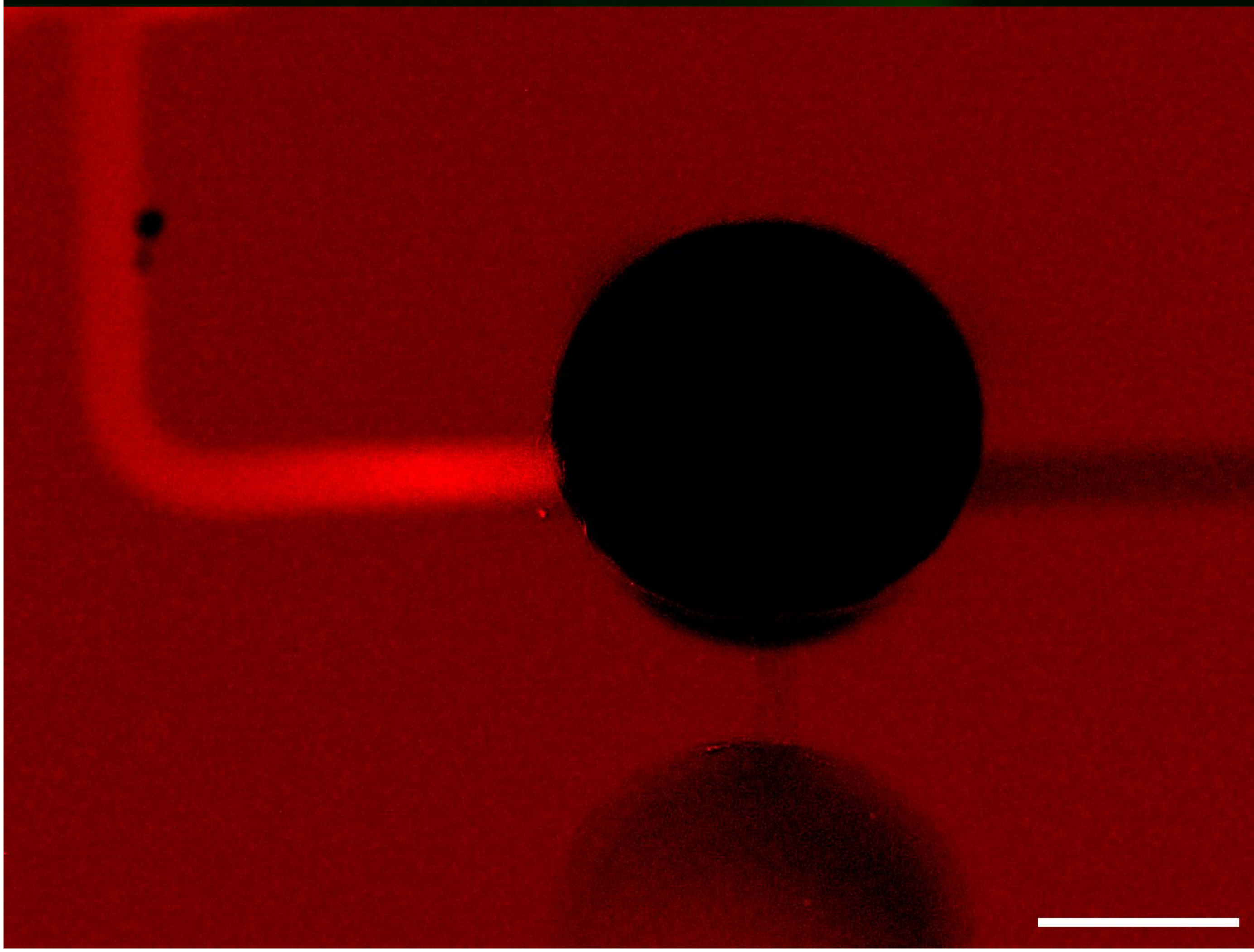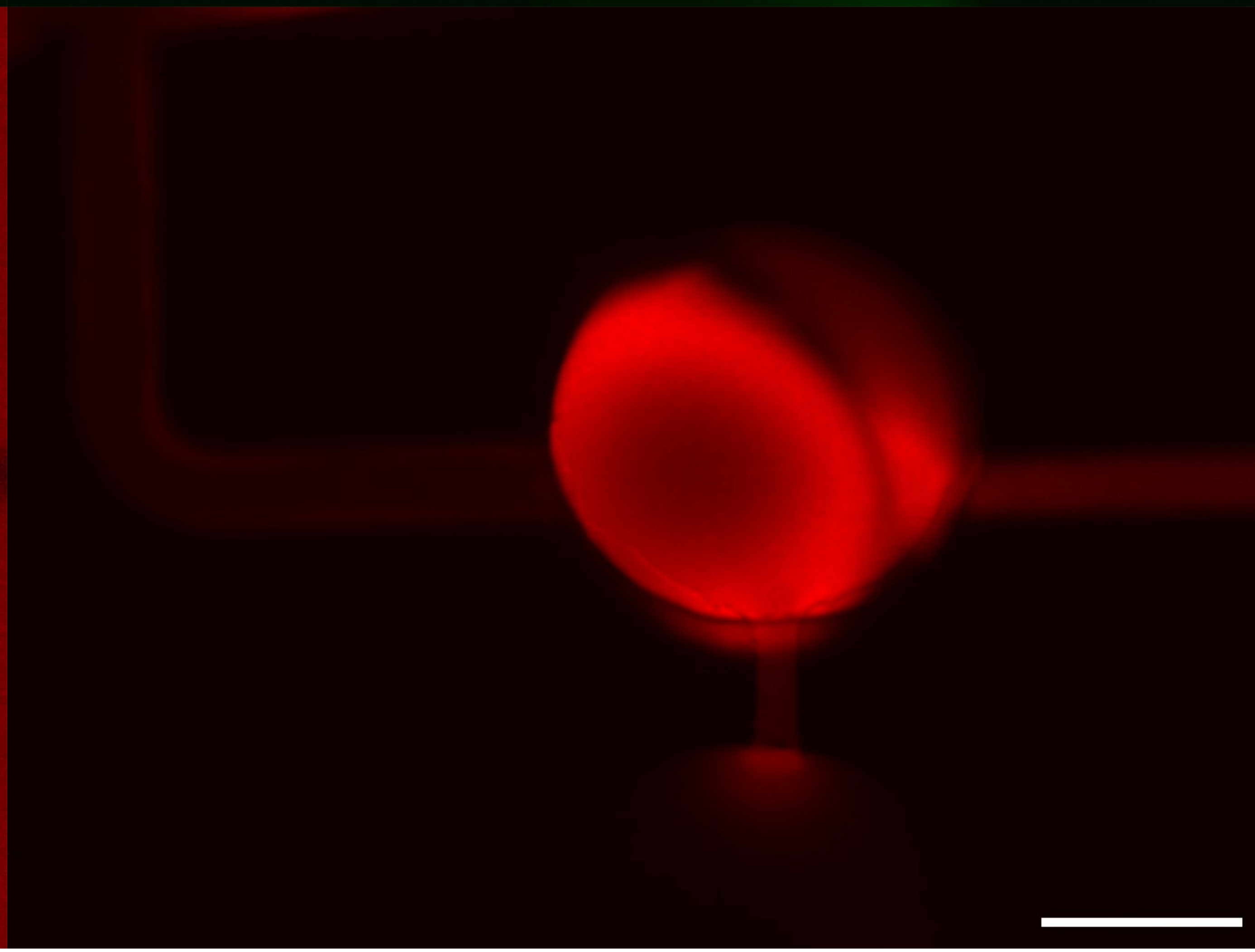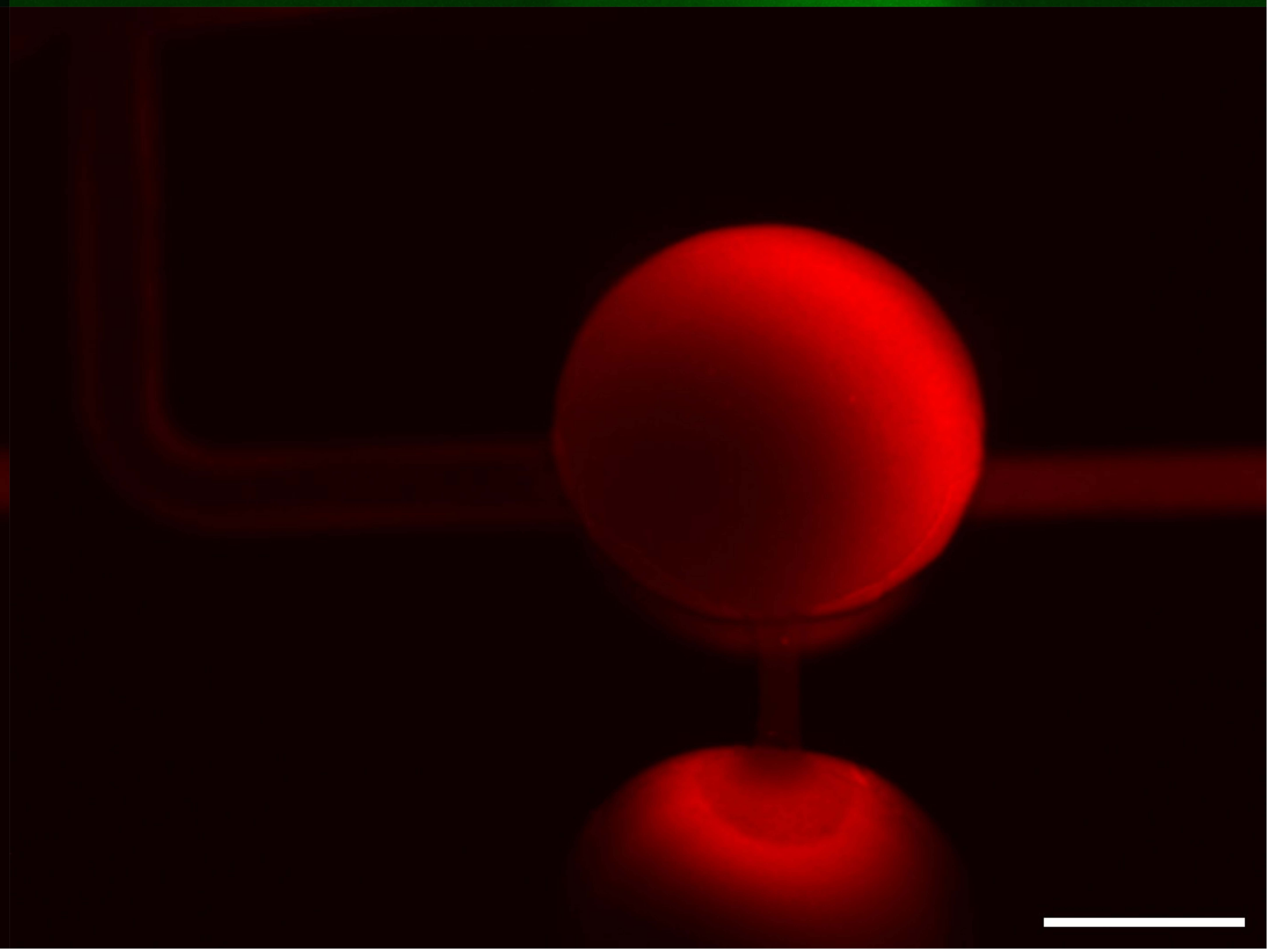

Supplement: Supplementary file 5 — SI Figure 4 [file 41378_2022_406_MOESM5_ESM.pdf]
